# Virological and care outcomes of community ART distribution: Experience with the PODI+ model in Kinshasa, Democratic Republic of the Congo

**DOI:** 10.1371/journal.pgph.0002343

**Published:** 2024-01-31

**Authors:** Michelle M. Gill, Winnie Bakebua, John Ditekemena, Cady Nyombe Gbomosa, Dieudonné Tshishi, Aimé Loando, Abhigya Giri, Roger Beni Ngantsui, Heather J. Hoffman

**Affiliations:** 1 Elizabeth Glaser Pediatric AIDS Foundation, Washington, District of Columbia, United States of America; 2 Elizabeth Glaser Pediatric AIDS Foundation, Kinshasa, Democratic Republic of the Congo; 3 Population Health Program, University of Ottawa, Ottawa, Canada; 4 Department of Biostatistics and Bioinformatics, Milken Institute School of Public Health, The George Washington University, Washington, District of Columbia, United States of America; 5 DRC Ministry of Health, National AIDS Control Program, Kinshasa, Democratic Republic of the Congo; Human Sciences Research Council, SOUTH AFRICA

## Abstract

**Introduction:**

Differentiated service delivery models for HIV treatment can minimize unnecessary burdens on health systems and promote efficient delivery of antiretroviral therapy (ART). Under the PODI+ (poste de distribution communautaire) model, ART multi-month dispensation (MMD) was provided by lay workers (peers) in communities. We compared outcomes among clinically stable adults living with HIV receiving MMD via PODI+ or health facility (HF).

**Methods:**

Clients receiving MMD at nine HFs and two PODI+ sites in Kinshasa were followed prospectively for one year (2018–2020). Medication possession ratio (MPR) was measured as proportion of total days with medication during the study through record abstraction at 3-month intervals. Viral load was assessed at enrollment and 12 months. We compared MPR and viral load suppression by arm and examined associations and potential confounders using unadjusted and adjusted odds ratios (AOR). Likert-style client satisfaction was collected during 12-month interviews and described by arm.

**Results:**

Odds of maintaining viral load suppression at 12 months for PODI+ participants were two times that for HF participants. In adjusted models, PODI+ participants had 1.89 times the odds of being suppressed at 12 months compared to HF participants (95% CI: 1.10, 3.27). No significant differences in MPR were found between groups (OR: 0.86, 0.38–1.99). Older participants had significantly higher odds of MPR (AOR: 1.02, 95% CI: 1.01, 1.03) and viral suppression (AOR: 1.03, 95% CI: 1.00, 1.07). Satisfaction with services was ≥87% overall, but PODI+ participants rated time spent at site, provider attributes and other care aspects more favorably.

**Conclusions:**

Participants receiving MMD via peer-run community distribution points had similar MPR, but better virological outcomes and greater satisfaction with care than clinically similar participants receiving MMD through facilities. PODI+ could be a useful model for expansion to serve larger clinic populations from overburdened health facilities, particularly as policy shifts towards more inclusive MMD eligibility requirements.

## Introduction

Scale-up of universal antiretroviral therapy (ART) has dramatically increased the number of people living with HIV (PLHIV) on treatment from an estimated 7.8 million in 2010 to 29.8 million in 2021, resulting in reduced morbidity and mortality among PLHIV [1]. Two-thirds of 39 million PLHIV worldwide live in sub-Saharan Africa (SSA), where health facilities are overburdened and the health workforce shortage is severe [1, 2]. While ART scale-up has been largely successful in SSA, viral suppression rates were 74% in eastern and southern Africa and 73% in West and Central Africa [3]. To meet UNAIDS 95-95-95 targets by 2025, particularly the second and third 95’s (95% of PLHIV receiving treatment and 95% of those receiving treatment with a suppressed viral load (VL), tailored service models are needed to more effectively address the unique needs of PLHIV to optimize their care and treatment.

Differentiated service delivery (DSD) models have been widely promoted as client-centered approaches that adapt HIV services to meet individual needs and minimize burdens on health systems. DSD models for HIV treatment can facilitate more efficient and effective ART dispensation through individual, group, or community-based delivery systems that reduce frequency of clinic visits, relocate services, and other strategies [4, 5]. Models are typically divided into four types: healthcare worker-managed groups, client-managed groups, facility-based individual models, and out-of-facility individual models [6]. In addition to reducing client burden at facilities, these models aim to increase retention in care, ART adherence, and viral suppression by addressing environmental, structural and social barriers, such as transportation costs and accessibility, provider attitudes and overall reception, stigma associated with clinic attendance, and service waiting times [7, 8].

In most countries, a variety of DSD models are now being implemented, however, client outcomes are often not captured by current medical record systems [9] and evidence is limited on whether DSD models result in equivalent or improved clinical outcomes compared to standard of care (SOC) [10]. A 2020 systematic review of DSD models for HIV treatment found that only 28% of included studies provided a comparison with either conventional care or another model, making it challenging to draw conclusions on the overall impact of DSD on clinical outcomes [10]. While there is growing evidence that models such as multi-month ART dispensation (MMD), home-based delivery, adherence groups, and community ART groups are at least non-inferior to standard care in SSA [6, 11–18], few studies have focused on community ART distribution models in which non-medical personnel provide both drug dispensation and other related services.

In 2010, Médecins Sans Frontières (MSF) introduced a DSD model in Kinshasa, the Democratic Republic of the Congo (DRC), to decentralize ART delivery for stable clients through community-based drug refill sites [19]. These sites, called “poste de distribution communautaire” (PODI), were staffed by expert client volunteers in an attempt to set apart drug provision from medical care [19]. A 2017 study of PODI client outcomes showed promising results for client ART retention and adherence with low rates of attrition [19]. In 2016, with support from the U.S. Agency for International Development (USAID), the Elizabeth Glaser Pediatric AIDS Foundation (EGPAF) expanded on MSF’s PODI model in two health zones in Kinshasa. The “PODI+” model shifts service delivery to a lay cadre of staff, peers also living with HIV, who provide ART refills and broader health services including TB screening, provision of Isoniazid preventive latent TB therapy and cotrimoxazole, nutrition and adherence counselling, support group facilitation, and family planning as well as tracing activities for those who miss visits. To understand the effect of the PODI+ model on continued client care, we conducted a prospective cohort study to compare the outcomes of clinically stable participants living with HIV receiving MMD via PODI+ sites or through facility-based care during a one-year period.

### HIV in the DRC

As of 2021, there were 540,000 people living with HIV in DRC, an estimated 82% of PLHIV knew their status, and 82% were receiving treatment [20]. According to the 2015 census, national treatment coverage was 33%, with only 3% of HIV patients on treatment receiving a VL test. In the country’s capital of Kinshasa, the HIV prevalence was 1.6%, almost double the national prevalence (0.8%) [21]. Alongside the HIV burden in DRC, there are barriers to treatment, such as ART stockouts, which can increase rate of interruptions in treatments, ART resistance, treatment attrition, and increased morbidity and mortality [22].

## Methods

### Study setting and site selection

The study took place in the only two EGPAF-supported PODI+ sites in Kinshasa and nine EGPAF-supported health facilities located in the Binza Meteo and Bandalungwa Health Zones (HZ); all study sites were located in semi-rural areas of the city. These HZ were identified for the comparison arm as they did not have PODI+ sites nor plans to establish them and they were located a considerable distance from the PODI+ sites to minimize risk of crossover between intervention and comparison arms. Study health facilities were purposively selected to represent a range of ART client volumes.

### Description of the study arms

Under the PODI+ model, MMD ART and related services were primarily delivered by peers, regularly supervised lay workers who received training in ART dispensation and counselling and other PODI+ services and mentorship from clinical staff. Annually, PODI+ clients typically underwent VL testing at the referral health facility or when a clinician visited the PODI+ site. Under the health facility model, MMD ART was provided along with counselling by peers and other health-related services as needed at each quarterly or semi-annual visit, also with yearly VL monitoring.

In both arms, drugs were typically supplied approximately every 3 to 6 months to clinically stable clients. Per the national definition at the time, clinically stable criteria included the following: ≥15 years of age, ≥12 months on ART, adherent to ART per provider assessment, no current opportunistic infections (OI), HIV VL suppression (VLS) defined as a result of <1,000 copies/mL within the last 12 months, and not pregnant [23]. Providers would offer MMD to clients meeting criteria; those receiving services in a HZ that also had a PODI+ site, were offered the option to receive MMD at the same health facility (HF) where they were currently receiving treatment or transfer to PODI+ for MMD. If clients no longer met MMD eligibility criteria–for instance due to VL rebound, OI development, poor adherence or becoming pregnant–they were to be switched back to SOC. This includes a return to HF for PODI+ clients, one-month refills, and additional care and treatment services as applicable (e.g., adherence-boosting sessions, TB treatment). If the issue was resolved, clients who were again eligible could return to MMD and PODI+ clients could resume care at the community site. Towards the end of the study enrollment period, dolutegravir (DTG) for adults was introduced around April 2019 and reached most eligible clients in EGPAF-supported sites in Kinshasa, including study sites, by September 2019.

### Data collection procedures

Clients receiving HIV services at study sites and potentially eligible for the study were referred by health providers to study staff for screening and recruitment. Participants had to meet clinical stability criteria as described above, with slight adjustments to reflect the practice in study sites (e.g., age ≥18 years and suppressed VL result within the past 12 months). They also had to be willing and able to provide informed consent and plan to stay in the study area for the next year. All participants provided written informed consent prior to the conduct of study procedures in French or Lingala (per their preference). Study enrollment took place between September 11, 2018 and June 13, 2019.

Participants were interviewed by study staff using a structured guide at enrollment and at six- and 12-months of study follow-up. The enrollment interview captured demographic data, HIV/ART history, side effects, and duration on MMD. At six and 12 months, we collected data on satisfaction with services, ART changes since last visit and other information. At approximately 3-, 6-, 9- and 12-months post-enrollment, data were abstracted from clinic records to capture information related to clinic visit attendance including switches off of MMD, any ART changes, ART scheduled and actual refill dates and doses dispensed at refill visit. Interim visit data was also abstracted; this primarily included visits to initiate participants on DTG-based ART. Provided there was sufficient drug stock, ART clients were typically first provided with two two-week refills of DTG for close clinical monitoring. If there were no issues identified, MMD clients would then transition back to their previous schedule (e.g., three months). Finally, dried blood spot samples were taken by venepuncture at study enrollment and approximatively 12 months after to assess VL using polymerase chain reaction testing. The endpoint was extended to 15 months to maximize VL availability, particularly as the ongoing COVID-19 pandemic made final participant follow-up more challenging. Any interim VL collected as part of routine services was not captured, but all switches off MMD to SOC due to unsuppressed VL and other reasons were documented as part of study follow-up.

Data were collected on paper-based study forms and entered using Epi-Info (v2.7; Atlanta, GA, USA). All data collection forms were reviewed and validated by the study team before entry into the database; additional quality assurance was performed following data entry. This study was approved by the University of Kinshasa School of Public Health Ethics Committee and Advarra Institutional Review Board in the United States (US). We also received administrative authorization from the HZ involved in the study and clearance from the community organizations which managed each PODI+ site and represented people living with HIV. Additional information regarding the ethical, cultural, and scientific considerations specific to inclusivity in global research is included in the S1 Checklist.

### Data analysis

Participant characteristics, HIV history and client satisfaction were summarized using frequencies and percentages and medians and interquartile ranges (IQR). Baseline characteristics were assessed for differences between interventions using Pearson’s chi-square tests for categorical variables and Wilcoxon rank sum tests for continuous variables. We compared two primary outcomes across comparison and intervention arms: medication possession ratio (MPR) and VLS at 12 months. Outcomes were assessed using an intent-to-treat approach; if participants in either arm switched back to SOC due to no longer meeting MMD criteria for some or all of their remaining follow-up, they were still classified in the same arm as baseline. Sensitivity analyses were conducted to address missing VL data at endline in which we assumed the following imputation scenarios at endline: suppressed, unsuppressed, the same VL outcome as at baseline (suppressed or unsuppressed), or the opposite VL outcome at baseline.

MPR was measured by the proportion of days with medication within the 12-month study period. We calculated proportion of days with medication within the approximate 12-month study period using participant drug pick-up dates and doses dispensed at each visit. Participants having drug possession ratios ≥90% were defined as adherent. We estimated the proportion of participants achieving VLS at enrollment and 12 months post-enrollment in each arm and associated 95% confidence intervals (CI).

We estimated the effect of PODI+ on MPR and VLS using odds ratio (OR) and 95% CI and accounted for clustering. Unadjusted and adjusted generalized estimating equations were used to examine the association between study arm and potential confounders for each outcome using a binomial distribution with a logit link function and compound symmetry working correlation structure to account for clustering of participants at multiple sites. Variables included in the models were determined by the literature and selected from any differences in enrollment characteristics that were also associated with the outcome (p-value <0.1) in unadjusted analyses. For any pair of independent variables that were highly associated with each other, only one variable was selected to be included in the multivariable model. Data were analyzed using SAS (v9.4; Cary, NC, USA).

## Results

### Screening and enrollment

**Fig 1:**
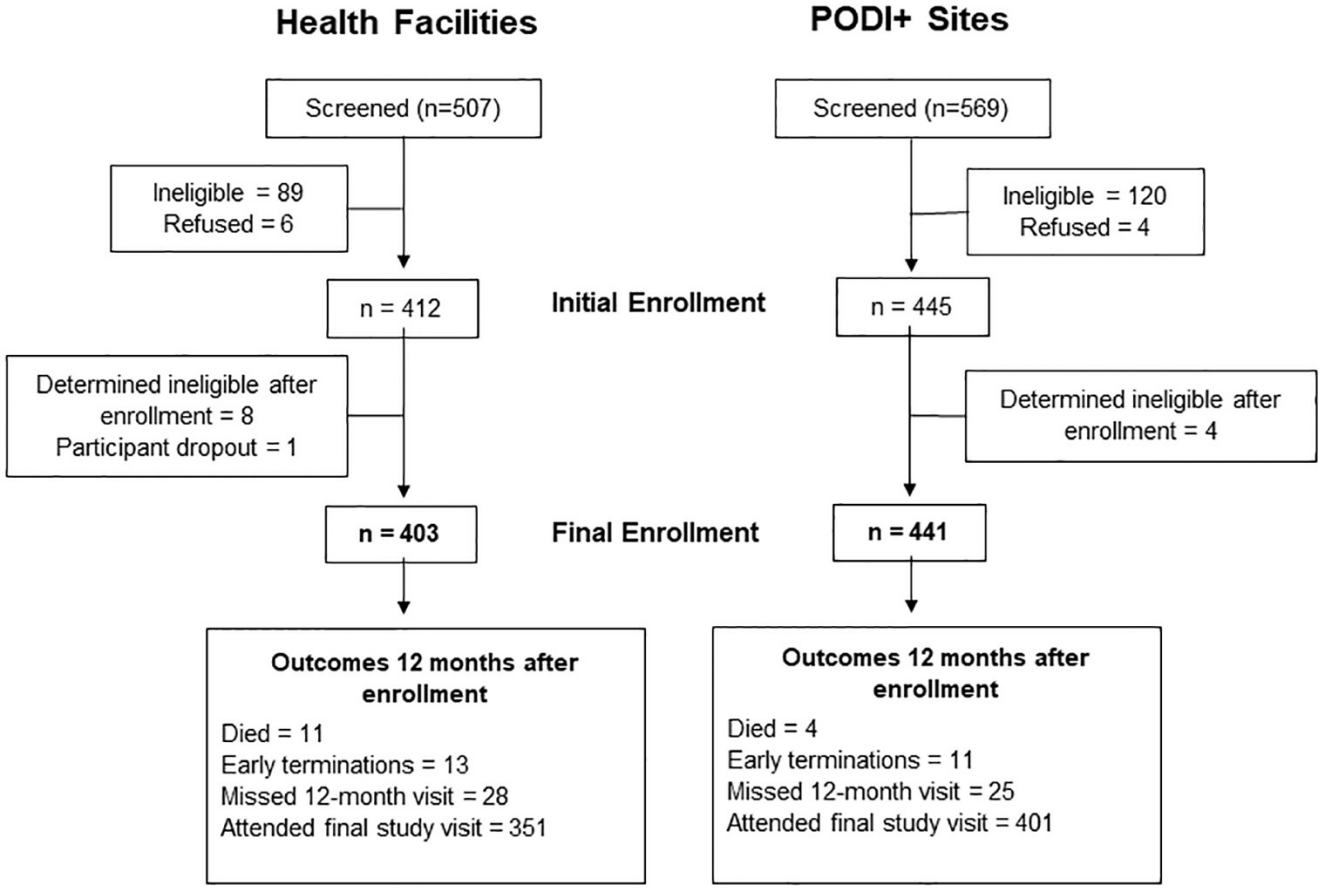
Screening, enrollment and follow-up by arm.

Overall, 1,076 clients were recruited at the 11 study sites (Fig 1): 507 in the HF arm and 569 in the PODI+ arm. In the two arms respectively, 403 and 441 eligible participants were enrolled, for a total of 844. Twelve clients were initially enrolled who did not meet study criteria, but they were terminated at their next clinical visit. Their data were not included in the database and the instances were reported to the ethics boards in the DRC and US.

Overall, the median age of participants was 48 years [IQR 40–55]); the majority (72.3%) were female (Table 1). They were on ART for a median of 5.4 years (IQR: 2.8–8.6). The majority of participants (92.2%) were on EFV-based ART at enrollment; three HF participants enrolled in May/June 2019 were on DTG ART. There were no differences in arms between viral suppression (<1,000 copies/ml) and unsuppressed (≥1,000 copies ml) at enrolment (p = 0.29). Baseline characteristics that differed significantly between groups were age (p = 0.0017), time on ART (p<0.0001), current ART regimen (p<0.0001) and MMD duration (p<0.0001). Duration was defined as time receiving care at site for the PODI+ arm, since all PODI+ clients were on MMD, and time on MMD for the HF arm. Most participants (87.7%) received three-month refills, though participants primarily in one health facility received drugs every two months and six (0.7%) HF participants received drugs every 4–6 months.

**Table 1:** Enrollment characteristics of study participants by intervention arm.

Characteristic	HF Participant (n = 403)	PODI+ Participant (n = 441)	p-value	Total (n = 844)
**Socio-Demographics**				
**Age, n, median years (IQR)**	**403, 47 (38–54)**	**441, 49 (43–56)**	**0.0017**	**844, 48 (40–55)**
≤ 24, n (%)	11 (2.5)	4 (0.9)		15 (1.8)
25–34	53 (13.2)	32 (7.3)		85 (10.1)
35–49	179 (44.4)	190 (43.1)		369 (43.7)
50+	160 (39.7)	215 (48.8)		375 (44.4)
**Sex, n (%)**			0.42	
Female	286 (71.0)	324 (73.5)		610 (72.3)
**Current marital status, n (%)**			0.66	
Married/co-habitating	164 (40.7)	173 (39.2)		337 (39.9)
Never married/ divorced/separated/widowed	239 (59.3)	268 (60.8)		507 (60.1)
**Educational attainment, n (%)**			0.29	
No education	11 (2.7)	17 (3.9)		28 (3.3)
Attended primary school	64 (15.9)	84 (19.1)		148 (17.5)
Attended secondary school or above	328 (81.4)	340 (77.1)		668 (79.1)
**HIV/ART-Related History**				
**Disclosed HIV status to, n (%)**			0.12	
No one	112 (27.8)	85 (19.3)		197 (23.3)
≥1 person(s), e.g., partner	291 (72.2)	356 (80.7)		647 (76.7)
**Time on ART, n, median years (IQR)**	**403, 4.8 (2.2–8.3)**	**441, 5.9 (3.3–9.1)**	**<0.0001**	**844, 5.4 (2.8–8.6)**
**Current ART regimen, n (%)**			**<0.0001**	
EFV ART	363 (90.1)	415 (94.1)		778 (92.2)
NVP ART	13 (3.2)	26 (5.9)		39 (4.6)
LPV/r ART	24 (6.0)	0		24 (2.8)
DTG ART	3 (0.7)	0		3 (0.4)
**Viral load result, enrollment**			0.29	0.29
<1,000 copies/ml	347 (89.7)	408 (93.4)		755 (91.6)
≥1,000 copies ml	40 (10.3)	29 (6.6)		69 (8.4)
Missing	16	4		20
**Duration of MMD, n, median, months (IQR)**	**403, 7.4 (2.0–20.4)**	**435, 17.5 (9.2–22.2)**	**<0.0001**	**838, 12.7 (5.0, 21.6)**
≤1 year, n (%)	246 (61.0)	152 (34.9)		398 (47.5)
>1–2 years	81 (20.1)	231 (53.1)		312 (37.2)
>2–5 years	47 (11.7)	52 (12.0)		99 (11.8)
>5 years	29 (7.2)	0		29 (3.5)
Missing	0	6		6

### Study follow-up

There were 24 total early terminations during study follow-up. These were among participants no longer in care at study sites (11 in each arm) or due to client refusal for further study participation (2 in HF arm). There were 15 deaths: 11 HF (2.7%) and 4 PODI+ (0.9%); causes of death were not consistently recorded. During study follow-up, there were 42/844 (5.0%) who switched off of MMD for at least part of study follow-up. Median months off of MMD was 7.5 (IQR: 5.9–9.2) and 6.2 (IQR: 4.5–8.33) among PODI+ and HF participants respectively who switched off MMD for any length of time. More HF participants switched compared to PODI+ participants in total (25/403 versus 17/441) and for reasons of unsuppressed VL or poor adherence specifically (23 versus 13). The difference was not significant (p = 0.10). Other reasons included developing TB, pregnancy, and excessive weight loss.

### Primary outcome analysis

#### MPR

Overall, 783/828 (94.6%) participants transitioned to DTG (411 PODI+, 372 HF) by September 30, 2019, excluding 16 who terminated before this date; 2.2% switched to DTG after this date and 2.8% had no documentation of switching during the study. During the approximate 12-month study period, 557/803 (69.4%) participants had ART in their possession at least 90% of the time; only 2.2% always had a supply of ART. Participants who died or terminated early (n = 39) did not contribute data to the model as they were missing DTG duration; two additional participants were also missing data. Accounting for clustering, the difference between PODI+ and HF participants with at least 90% MPR was not statistically significant (69.8% vs. 68.9%, OR 1.05 [95% CI: 0.34, 3.17]).

In a multivariable model, PODI+ participants had a non-significantly lower odds (OR: 0.86, 95% CI 0.38–1.99, p = 0.73) of ≥90% MPR compared to HF participants (Table 2), after adjusting for age, HIV disclosure status, gender, duration on DTG, and duration on MMD. Time on ART and current ART regimen were excluded from the model due to collinearity with MMD duration. Older participants had significantly higher odds of ≥90% MPR (AOR: 1.02, 95% CI: 1.01, 1.03) after adjusting for the other variables.

**Table 2:** Odds of ≥90% MPR versus <90% MPR from unadjusted and adjusted generalized estimating equations.

Variable	N (%)	Unadjusted			Adjusted		
		OR	95% CI	p-value	OR	95% CI	p-value
**Intervention Arm**							
PODI+	441 (52.3)	0.89	0.36, 2.20	0.8062	0.86	0.38, 1.99	0.73
HF	403 (47.8)						
**Age at enrollment**	--	1.02	1.01, 1.03	**<0.0001**	1.02	1.01, 1.03	**<0.0001**
**Gender**							
Male	234 (27.7)	1.02	0.79, 1.31	0.9047	0.99	0.76, 1.30	0.95
Female	610 (72.3)						
**HIV status disclosure**							
No one	197 (23.3)	1.35	0.93, 1.97	0.1182	1.31	0.89, 1.93	0.17
≥1 person(s)	647 (76.7)						
**Duration on DTG**							
<6 months	490 (63.0)	0.92	0.64,	0.68	0.91	0.61,	0.65
≥6 months	288 (37.0)		1.34			1.36	
**Duration on MMD**							
<1 year	398 (47.5)	1.09	0.87, 1.35	0.47	0.98	0.78, 1.22	0.83
≥1 year	440 (52.5)						

#### Viral load suppression

At the study end point, VLS in the PODI+ arm was 97.0% (390/402) compared to 93.6% (339/362) in the HF arm. Odds of maintaining VLS at approximately 12 months for PODI+ participants were two times that for HF participants (OR: 2.21, 95% CI: 1.00, 4.84). Taking VL coverage into account by including all those who were still in the study but did not have final VL monitoring, rates were 91.5% (390/426) and 89.4% (339/379), respectively; the difference was not significant (OR: 1.28, 95% CI: 0.49, 3.36). To determine the odds of maintaining VLS in a multivariable model (Table 3), we only included those who had an enrollment and final VL, 97.0% (387/399) and 93.4% (324/347) in the PODI+ and HF arms, to account for the possible effect of enrollment VL on the final outcome. In this model, we found PODI+ participants had a significantly higher odds (AOR: 1.89, 95% CI: 1.10, 3.27) of being suppressed at 12-months compared to HF participants, after adjusting for baseline VL, age, disclosure status, gender, DTG duration, and duration of MMD. Older participants also had significantly higher odds of being suppressed at 12 months (AOR: 1.03, 95% CI: 1.00, 1.07) after adjusting for the other variables. A sensitivity analysis that involved additional imputation of missing values for VL results at endline remained significant and did not change the direction of the relationship.

**Table 3:** Odds of being suppressed versus unsuppressed at 12-months from unadjusted and adjusted generalized estimating equations.

Variable	N (%)	Unadjusted			Adjusted		
		OR	95% CI	p-value	OR	95% CI	p-value
**Intervention Arm**							
PODI+	441 (52.3)	2.27	1.18, 4.38	**0.0140**	1.89	1.10, 3.27	**0.022**
HF	403 (47.8)						
**Age at enrollment**	—	1.02	1.01, 1.04	**0.0075**	1.03	1.00, 1.07	**0.045**
**Gender**							
Male	234 (27.7)	0.87	0.56, 1.35	0.5344	0.85	0.44, 1.65	0.63
Female	610 (72.3)						
**HIV status disclosure**							
No one	647 (76.7)	1.08	0.71, 1.64	0.7086	1.07	0.51, 2.23	0.87
≥1 person(s)	197 (23.3)						
**Duration on DTG**							
<6 months	490 (63.0)	1.60	1.05, 2.42	**0.028**	1.32	0.73, 2.39	0.36
≥6 months	288 (37.0)						
**VL at enrollment**							
Suppressed	755 (91.6)	1.87	0.59, 5.90	0.2887	1.04	0.29, 3.70	0.96
Unsuppressed	69 (8.4)						
**Duration on MMD**							
<1 year	398 (47.5)	2.45	1.16, 5.15	**0.018**	2.07	0.78, 5.49	0.15
≥1 year	440 (52.5)						

### Client satisfaction

**Fig 2:**
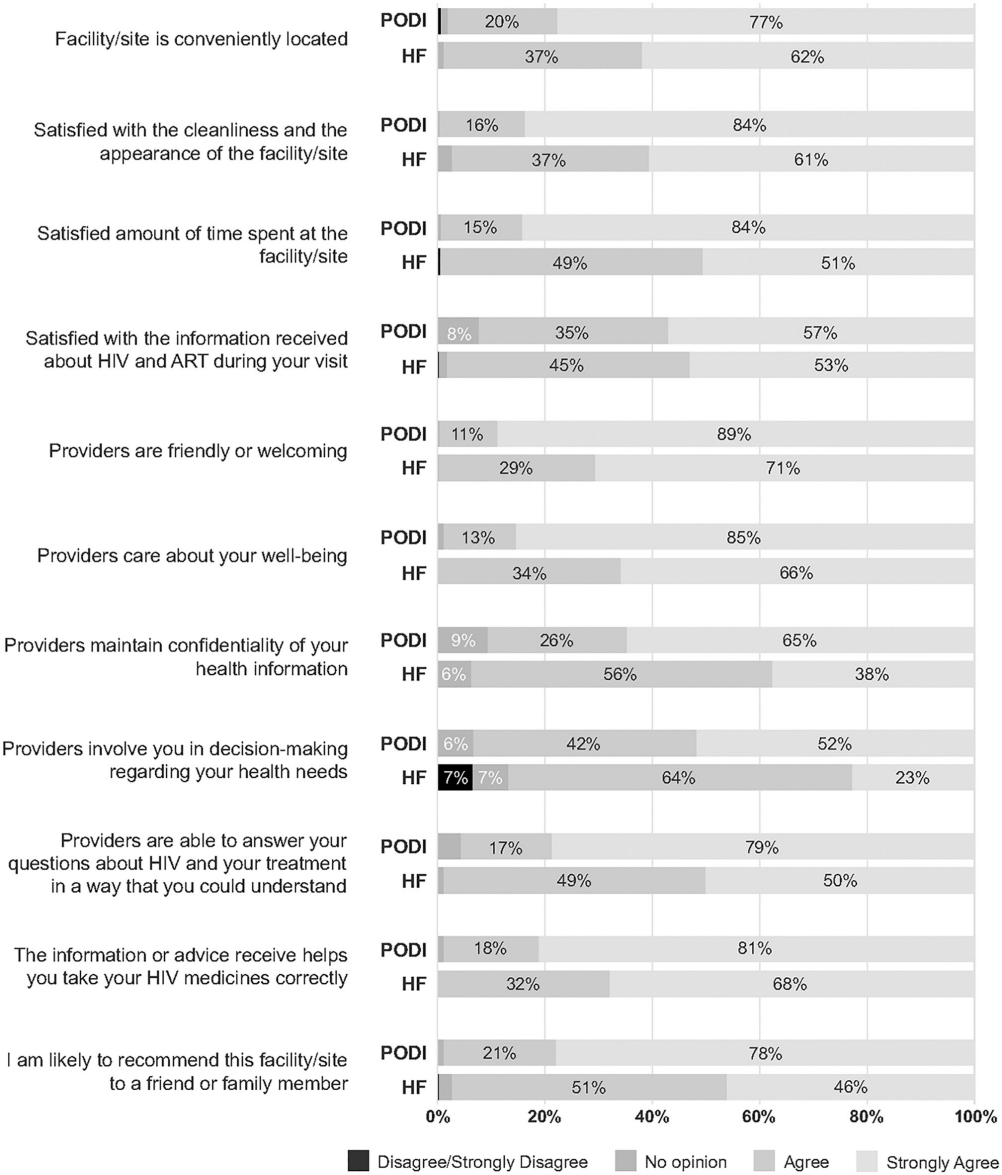
Satisfaction with care at 12-month interview, by intervention arm.

HF (n = 330) and PODI+ (n = 402) participants were asked about aspects of service quality as part of the 12-month interview (Fig 2). Participants in both arms had high levels of satisfaction; ≥87% agreed or strongly agreed with statements. However, those in the PODI+ arm consistently expressed a higher degree of satisfaction than the HF arm. The largest differences between agree and strongly agree were as follows: satisfied with amount of time spent at site (84% PODI+ vs. 51% HF), providers maintaining confidentiality (65% vs. 38%), provider answers health questions in a way the client can understand (79% vs. 50%), and likelihood of recommending site to someone they know (78% vs. 46%). PODI+ participants also viewed other provider attributes more favorably than HF participants: 18% more strongly agreed that providers were friendly or welcoming and 19% more strongly agreed that providers cared about client well-being. The only statement that more than a few participants disagreed with, was ‘providers involve you in decision-making regarding your health needs;’ 22 (6.7%) HF participants disagreed compared to one PODI+ participant.

## Discussion

Clinically stable participants living with HIV who received ART MMD through PODI+ sites primarily run by peers did not have significantly different times without medication, but had better virological outcomes compared to participants receiving MMD through HF. Older age was significantly associated with both MPR and VLS. While satisfaction with services was generally high across both arms, PODI+ participants tended to rate their quality of care more favorably than those at HFs, particularly in terms of satisfaction with amount of time spent at site and provider attitudes and interactions. This study provides comparative evidence of two clinically similar groups of clients receiving MMD through facilities and a unique intervention that expands upon community ART distribution by offering limited other HIV and related services.

Several other studies assessing retention and viral suppression between facility and community MMD approaches among clinically stable clients living with HIV have similarly found these outcomes to be non-inferior or better in community settings versus SOC [12, 15, 17, 18, 24]. Most studies looked at retention based on 12-month endpoints [12, 15, 16, 18, 24, 25]. Our study used MPR to take actual doses dispensed into account. This has been used in other MMD research, though a study of adherence club and SOC beneficiaries in Zambia found a higher MPR than in our study; 96–100% had ART in their possession [7] compared to 69% who possessed treatment at least 90% of the time in our study. A review of DSD models found retention similar across arms and slight increases in VLS in the intervention arm [10]. Many of these studies also noted missing data, particularly for VL as a limitation [7, 12, 15, 17, 24, 26, 27]. About 5% of participants completed follow-up in our study but did not contribute VL data. This was likely due in part to COVID-19 pandemic policy-imposed movement restrictions and individual responses to reduce potential exposures.

While most studies have focused on community ART refill groups, adherence clubs and home/mobile ART delivery, there is limited comparative evidence assessing MMD through a community ART distribution site offering other additional limited services. This comparative evaluation builds off of earlier work which described outcomes of the initial PODI model, primarily ART dispensation plus counselling [19, 28, 29]. Deaths among PODI+ participants (0.9%) in our study fell between previously reported rates of 0.2% and 1.5% in these prior PODI evaluations; we also found a lower rate of PODI+ participants switching back to HF SOC (<4%) compared to 8% of PODI clients, though the follow-up period was not specified [28]. In a similar evaluation, clients had significantly better retention after 12 months of receiving MMD through PODI+ and other differentiated care models compared to facility-based delivery [30].

Older adults were overrepresented in our study population, especially in the PODI+ arm, with only 12% of participants < 35 years of age. We found slightly elevated odds of better MPR and VLS outcomes among older participants. Other studies found retention in adherence clubs and out-of-facility MMD increased with older age among beneficiaries [16, 18]. More generally, older age has been associated with better health outcomes than those experienced by younger PLHIV, including ART adherence, retention, and viral suppression, often among clients ≥40 years of age [31–34]. Older adults may have had more opportunity or greater interaction with the health system to develop effective HIV management strategies or place more value on health promotive behaviors. Other factors, such as experiencing greater stabilization in one’s life or fewer pressures than their younger counterparts to engage in behaviors leading to non-adherence have also been cited [32, 34].

While there was general satisfaction with services found across arms, PODI+ participants expressed a greater degree of satisfaction than HF participants. This could have a sustained effect on longer-term care received under this model, particularly the aspects of care addressing convenience and caring and trusted provider relationships. High satisfaction has also been found in home ARV delivery by lay workers and adherence clubs led by pharmacists supported by community workers [7, 27]. As in our study, Roy et al found one aspect contributing to client acceptability was patient-centeredness. Clients initiating ART and refilling medication through mobile units achieved greater VLS than those receiving standard facility care. Reasons included informal and flexible communication between providers and clients, streamlined service delivery resulting in shorter visits compared to health facilities, and more time to provide comprehensive and tailored counselling as needed, endeared clients to their providers and encouraged healthy disease management [35].

While DSD models for HIV treatment have been widely rolled out across SSA for over decade, a rapid expansion of DSD models, particularly MMD and community dispensation occurred in 2020 in response to the COVID-19 pandemic [36–38]. These DSD models supported ART continuity while mitigating COVID-19 exposure at facilities. While our study and others described outcomes for clinically stable clients, the pandemic prompted policy shifts towards less restrictive models. More evidence is needed to understand how MMD and other DSD approaches perform among children and clients not meeting stability criteria. Emerging findings on DSD for newly initiating ART clients present a mixed picture on retention in care, with more favorable outcomes among clients receiving MMD immediately following facility or community ART initiation compared to community initiation and monthly dispensation [26, 39]. These outcomes may have been influenced by a shorter follow-up period and negative perceptions of lay worker administered treatment.

This study has notable limitations. First, we did not restrict study eligibility to clients newly starting MMD or otherwise standardize MMD duration for study participants. Therefore, participants had varying times on DSD, though we did account for this and other differences in multivariable models. Secondly, we only captured VL results that were collected as part of study baseline and endline, so we are not able to describe any interim changes. Baseline VL results were provided to study sites to inform clinical care, so it is unlikely that additional samples were collected during the year as part of routine annual VL monitoring. However, we did capture any instance of a switch from MMD to SOC if additional testing was performed in the interim due to suspected treatment failure that resulted in a change to care. Thirdly, while participants in both arms had similar durations off of MMD (about one month more in the PODI+ arm), PODI+ clients returned to their former HF to receive SOC, whereas the setting remained the same in the HF arm. Any misclassification bias would have been minimal as transfers for any length of time comprised only 5% of the overall cohort. Finally, participants were not randomized to an intervention arm which could have posed a selection bias. Providers offer PODI+ as an option to clients meeting eligibility criteria and clients may opt in. However, this study evaluated the program being implemented and reflected current practices at the time. Clients selecting the best mode of care for them (facility or community distribution) is a key tenet of DSD.

## Conclusions

Participants receiving MMD via peer-run community distribution points had similar medication possession ratios and better virological outcomes. With these similar or superior outcomes plus greater satisfaction with care than clinically similar participants receiving MMD through health facilities, the PODI+ model offers at least comparable or favorable service delivery. PODI+ could be a useful model for expansion to serve larger clinic populations from overburdened health facilities, particularly as policy shifts towards more inclusive eligibility requirements for MMD.

## Supporting information

S1 Checklist Inclusivity in global research. (DOCX)

S1 Data Dataset. (XLSX)
